# Dysfunctional interferon-α production by peripheral plasmacytoid dendritic cells upon Toll-like receptor-9 stimulation in patients with systemic lupus erythematosus

**DOI:** 10.1186/ar2382

**Published:** 2008-03-06

**Authors:** Seung-Ki Kwok, June-Yong Lee, Se-Ho Park, Mi-La Cho, So-Youn Min, Sung-Hwan Park, Ho-Youn Kim, Young-Gyu Cho

**Affiliations:** 1 Department of Medicine, Division of Rheumatology, Center for Rheumatic Diseases and Rheumatism Research Center, Catholic Research Institutes of Medical Sciences, Catholic University of Korea, Banpo-Dong, Seocho-Gu, Seoul, 137-701, Korea; 2 School of Life Sciences and Biotechnology, Korea University, Anam-Dong, Seongbuk-Gu, Seoul, 136-701, Korea

## Abstract

**Background:**

It is well known that interferon (IFN)-α is important to the pathogenesis of systemic lupus erythematosus (SLE). However, several reports have indicated that the number of IFN-α producing cells are decreased or that their function is defective in patients with SLE. We studied the function of plasmacytoid dendritic cells (pDCs) under persistent stimulation of Toll-like receptor (TLR)9 via a TLR9 ligand (CpG ODN2216) or SLE serum.

**Methods:**

The concentrations of IFN-α were determined in serum and culture supernatant of peripheral blood mononuclear cells (PBMCs) from SLE patients and healthy controls after stimulation with CpG ODN2216 or SLE serum. The numbers of circulating pDCs were analyzed by fluoresence-activated cell sorting analysis. pDCs were treated with CpG ODN2216 and SLE serum repeatedly, and levels of produced IFN-α were measured. The expression of IFN-α signature genes and inhibitory molecules of TLR signaling were examined in PBMCs from SLE patients and healthy control individuals.

**Results:**

Although there was no significant difference in serum concentration of IFN-α and number of circulating pDCs between SLE patients and healthy control individuals, the IFN-α producing capacity of PBMCs was significantly reduced in SLE patients. Interestingly, the degree which TLR9 ligand-induced IFN-α production in SLE PBMCs was inversely correlated with the SLE serum-induced production of IFN-α in healthy PMBCs. Because repeated stimulation pDCs with TLR9 ligands showed decreased level of IFN-α production, continuous TLR9 stimulation may lead to decreased production of IFN-α in SLE PBMCs. In addition, PBMCs isolated from SLE patients exhibited higher expression of IFN-α signature genes and inhibitory molecules of TLR signaling, indicating that these cells had already undergone IFN-α stimulation and had become desensitized to TLR signaling.

**Conclusion:**

We suggest that the persistent presence of endogenous IFN-α inducing factors induces TLR tolerance in pDCs of SLE patients, leading to impaired production of IFN-α.

## Introduction

Systemic lupus erythematosus (SLE) is a systemic autoimmune disease that is characterized by generation of autoantibodies against nuclear DNA and/or nuclear proteins [1]. The precise pathogenesis of SLE remains unknown, but both genetic and environmental factors are involved [2]. Over the past two decades numerous studies have suggested that interferon (IFN)-α may play a pathogenic role in SLE. This view is derived from the observation that IFN-α treatment in patients with nonautoimmune diseases, such as malignant tumors, induced anti-double-stranded DNA antibodies and occasionally resulted in the development of an SLE-like syndrome [3-7]. SLE patients exhibit ongoing IFN-α production, and IFN-α serum levels are correlated with both disease activity and severity [8,9]. IFN-α levels also correlate with anti-double-stranded DNA antibody production, complement activation, and IL-10 production, all of which are important indicators in SLE disease progression [9].

IFN-α plays a role in the activation, differentiation, and survival of B cells, T cells, and dendritic cells. IFN-α is mainly produced in plasmacytoid dendritic cells (pDCs); they were originally termed natural IFN-α producing cells [10-12]. pDCs are key effector cells in the innate immune system because of their ability to produce large amounts of IFN-α in response to microbial and viral infections. Human pDCs selectively express Toll-like receptor (TLR)7 and TLR9 within the endosomal compartment. These receptors are activated in response to viral RNA and DNA, leading to production of IFN-α [12,13]. Recent studies have shown that DNA-containing immune complexes within SLE serum stimulate pDCs to produce IFN-α [14,15], which is mediated cooperatively by TLR9 and FcγRIIa (CD32) [15].

Patients with SLE have reduced numbers of circulating natural IFN-α producing cells. The levels of IFN-α produced by SLE peripheral blood mononuclear cells (PBMCs) induced by SLE serum that contained an endogenous IFN-α-inducing factor, herpes simplex virus type 1, or the D type of CpG motif were lower than those produced by healthy control PBMCs, and the IFN-α producing capability of circulating pDCs in SLE patients may be impaired [16-18]. These results do not fit well with the role of IFN-α in SLE pathogenesis described above, although they suggest that the local concentration of IFN-α in an affected region is important. Furthermore, there have been reports that the pDCs from SLE patients and healthy control individuals produce similar amounts of IFN-α on a per cell basis in response to viral infection [17,19]. We wished to establish definitively the IFN-α production capability of PBMCs from SLE patients, and we show that IFN-α production is dysfunctional in pDCs from such patients.

We hypothesized that the persistent presence of DNA-containing immune complexes, which stimulate TLR9, affects the function of pDCs resulting in their malfunction. We analyzed serum levels of IFN-α in SLE patients and *in vitro *production of IFN-α in isolated PBMCs subjected to artificial stimulation by CpG ODN2216, which specifically activates human TLR9 in pDCs but not in B cells [20]. We also examined the number of circulating pDCs in SLE patients, as compared with those in healthy control individuals, using specific pDC surface markers and flow cytometry. The expression of IFN-α signature genes (IFN-α responsive genes) and inhibitory molecules of the TLR signaling cascade were examined. Our findings suggest that pDCs are dysfunctional in patients with chronic SLE, which is probably due to desensitization of TLR9 as a result of over-stimulation by DNA-containing immune complexes that are present in the sera of SLE patients.

## Materials and methods

### Patients and control individuals

This study was approved by the Institutional Review Board of Kangnam St. Mary's Hospital (Seoul, Korea) and all participants provided informed consent. Forty-three consecutive patients (two males and 41 females; age 35 ± 8.4 years) who presented at the rheumatology clinic and fulfilled the revised classification criteria for SLE [21] were enrolled in the study. Twenty-six volunteers (one male and 25 females; age 38.4 ± 4.3 years) were recruited to serve as healthy control individuals. Among the 43 patients, two patients were off prescription medication (in remission) and three were receiving prednisolone alone (mean dosage 12.5 mg/day; range 5 to 20 mg/day). The remaining 38 patients were receiving both prednisolone (mean dosage 13.3 mg/day; range 2.5 to 125 mg/day) and adjunctive therapies, such as hydroxychloroquine (34 patients; 200 or 400 mg/day), azathioprine (four patients), mizoribine (four patients), mycophenolate mofetil (three patients), and cyclosporine (one patient). Disease activity was scored using the SLE Disease Activity Index (SLEDAI) [22]. Descriptive statistics and clinical data for the SLE patients are described in Table 1.

**Table 1:** Demographic and clinical characteristics of SLE patients

Characteristic	SLE patients (n = 43)
Age (years; mean [range])	35.0 (21 to 56)
Sex (*n*; males/females)	2/41
Disease duration (years; mean [range])	8.5 (0.1 to 18)
SLEDAI (mean ± SD)	3.72 ± 3.84
Active (n = 9)^a^	9.55 ± 4.50
Inactive (n = 34)	2.17 ± 1.42

Cutaneous manifestation (n [%])	26 (60.5)
Arthritis (n [%])	27 (62.8)
Renal manifestation (n [%])	9 (20.9)
Cytopenia (n [%])	11 (25.6)
Serositis (n [%])	8 (18.6)
Prednisolone (*n *[%]; mean dosage [mg/day])	41 (95.3); 13.0
Hydroxychloroquine (n [%])	34 (79.1)
Azathioprine (n [%])	4 (9.3)
Cyclosporin (n [%])	1 (2.3)
Mizoribine (n [%])	4 (9.3)
Mycophenolate mofetil (n [%])	3 (7.0)

^a^Systemic Lupus Erythematosus Disease Activity Index above 4. SD, standard deviation; SLE, systemic lupus erythematosus.

### Preparation of PBMCs and pDCs

Blood samples obtained from patients and healthy control individuals were collected in heparinized tubes (BD Biosciences, San Jose, CA, USA), and PBMCs were prepared using Ficoll-Hypaque (Amersham Bioscience, Pascataway, NJ, USA) density gradient centrifugation. Cells were washed and suspended in RPMI 1640 medium (GibcoBRL, Carlsbad, CA, USA) containing penicillin (100 U/ml), streptomycin (100 μg/ml), and 10% fetal bovine serum (GibcoBRL) that had been inactivated by heating to 56°C for 1 hour. Healthy pDCs were isolated from PBMCs using a Diamond Plasmacytoid Dendritic Cell Isolation Kit (Miltenyi Biotec, Bergisch Gladbach, Germany); pDC purity was greater than 95%. The purified pDCs were cultured in RPMI 1640 medium containing 10% fetal bovine serum, granulocyte-macrophage colony stimulating factor (10 ng/ml), and IL-3 (10 ng/ml).

### Flow cytometry

PBMCs were incubated with human IgG to block the Fc receptor and then incubated with anti-CD123-PE-Cy5 monoclonal antibody (Mouse IgG_1_; BD Pharmingen™, San Jose, CA, USA), anti-BDCA-2-fluorescein isothiocyanate, and monoclonal antibody (Mouse IgG_1_; Miltenyi Biotec) for 30 minutes at 4°C; isotype control experiments were conducted in parallel. After two washes, the cells were re-suspended in phosphate-buffered saline and analyzed by flow cytometry. pDCs were identified by dual staining for both CD123 and blood dendritic cell antigen (BDCA)-2.

### IFN-α induction

PBMCs (1 × 10^6 ^cells) were stimulated using 1 μmol/l CpG ODN2216 (InvivoGen, San Diego, CA, USA) or 30% serum from SLE patients. Duplicate cultures were performed in 48-well plates (NUNK, Roskilde Denmark) at a final volume of 500 μl/well. After 24 hours IFN-α was measured from the supernatant.

### Measurement of IFN-α

Supernatants collected from sera and cell cultures were stored at -70°C until further use. The amounts of IFN-α in the sera and supernatants were then measured using a sensitive sandwich ELISA kit (Bender MedSystems, Vienna, Austria). A representative standard curve for the IFN-α ELISA is shown in Additional file 1 (Supplementary Figure 1). All measurements were performed in duplicate and averaged values were used in the data analysis.

### pDC stimulation with TLR9 ligand and SLE serum

Purified pDCs (2 × 10^4 ^cells) were incubated with or without the TLR9 ligand CpG ODN2216 (1 μmol/l; InvivoGen) or 30% serum from SLE patients in 96-well plates (NUNK) at a final volume of 200 μl/well. After 24 hours the pDCs were carefully washed with serum-free RPMI and re-treated with or without CpG ODN2216 (1 μmol/l) or 30% serum from SLE patients. The supernatants were harvested after an additional 24 hours and IFN-α production was measured using ELISA. In the recovery assay, pDCs were treated with 1 μmol/l CpG ODN2216 for 24 hours, washed with serum-free medium, cultured with serum-containing medium for 0, 24, or 48 hours, and then treated again with CpG ODN2216 (1 μmol/l). After 24 hours IFN-α production was measured from the supernatant.

### Cell viability assay

Relative cell viability was measured by Quick Cell Proliferation Assay kit (BioVision, Mountain View, CA, USA). Briefly, 1/10 volume of WST-1/electrocoupling solution were added into the culture media, incubated 4 hours in 5% carbon dioxide incubator, and measured the absorbance of the treated and untreated samples with water-soluble tetrazolium salt (WST)-1/electrocoupling solution using a microtiter plate reader at 450 nm. Each sample was duplicated and averages of the absorbance were used in comparisons. The differences in absorbance between treated and untreated samples was shown as relative cell viability.

### Reverse transcription PCR

Total RNA was extracted from isolated PBMCs or cultured cells using RNAzol B (Biotex Laboratories, Houston, TX, USA), in accordance with the manufacturer's instructions. Reverse transcription using 2 μg total RNA was carried out at 42°C using the Superscript reverse transcription system (Takara, Shiga, Japan). PCR amplification was performed in a reaction mixture containing 2.5 mmol/l dNTPs, 2.5 U Taq DNA polymerase (Takara), 0.25 μmol/l sense and antisense primers, and PCR buffer (1.5 mmol/l MgCl_2_, 50 mmol/l KCl, 10 mmol/l Tris-HCl [pH 8.3]). Reactions were processed in a DNA thermal cycler (Perkin-Elmer Cetus, Wellesley, MA, USA). PCR products were separated on a 2.5% agarose gel and stained with ethidium bromide. The latest cycle number during which PCR products were not yet saturated was selected and used to compare treatments using Quantity One software (version 4.5.2; BioRad, Hercules, CA, USA). Results are expressed as the ratio of target PCR products to GAPDH (glyceraldehyde-3-phosphate dehydrogenase) or β-actin product. The used primer pairs are described in Table 2.

**Table 2:** Sequences of primer pairs

Target molecules	Sequence
GAPDH	Forward: 5'-CGA TGC TGG GCG TGA GTA C-3' Reverse: 5'-CGT TCA GCT CAG GGA TGA CC-3'
β-actin	Forward: 5'-GGA CTT CGA GCA AGA GAT GG-3' Reverse: 5'-TGT GTT GGC GTA CAG GTC TTT G-3'
TLR9	Forward: 5'-GTG CCC CAC TTC TCC ATG-3' Reverse: 5'-GGC ACA GTC ATG ATG TTG TTG-3'
IFI44	Forward: 5'-CTC GGT GGT TAG CAA TTA TTC CTC-3' Reverse: 5'-AGC CCA TAG CAT TCG TCT CAG-3'
IFIT1	Forward: 5'-CTC CTT GGG TTC GTC TAC AAA TTG-3' Reverse: 5'-AGT CAG CAG CCA GTC TCA G-3'
PRKR	Forward: 5'-CTT CCA TCT GAC TCA GGT TT-3' Reverse: 5'-TGC TTC TGA C G GTA TGT ATT A-3'
MyD88s	Forward: 5'-CGG CAA CTG GAG ACA CAA G-3' Reverse: 5'-TCT GGA AGT CAC ATT CCT TGC-3'
IRAK-M	Forward: 5'-TTT GAA TGC AGC CAG TCT GA-3' Reverse: 5'-GCA TTG CTT ATG GAG CCA AT-3'

GAPDH, glyceraldehyde-3-phosphate dehydrogenase; IFI44, interferon-induced protein 44; IFIT1, interferon-induced protein with tetratricopeptide repeats 1; PRKR, Interferon-inducible double-stranded RNA-dependent protein kinase; TLR, Toll-like receptor.

### Measuring immune complex levels in SLE patients' sera

To measure the serum levels of immune complexes in SLE patients, we used the CIC-C1Q Circulating Immune Complexes ELISA kit (Bühlmann Laboratories AG, Schonenbuch, Switzerland). Circulating immune complexes from sera from patients with chronic SLE and control individuals were incubated with human C1q, which was adsorbed onto microtiter wells. After a washing step, we added alkaline phosphatase-conjugated protein A, which binds to the Fc region of human IgG. After an additional washing step, the enzyme substrate (paranitrophenyl-phosphate) was added, followed by a stop solution. The absorption at 405 nm of each sample was measured. We used 14 SLE sera, two of which were from patients with SLEDAI values of 12 and 16, and the remainder were from patients with SLEDAI values of less than 4. The two active SLE sera exhibited 1.8 U and 6.3 U of immune complexes.

### Statistical analysis

Differences between groups were analyzed using the Mann-Whitney U-test or Student's *t*-test. Correlation analyses were performed using Spearman's rank correlation test. All analyses were performed using SPSS (version 10.0; SPSS Inc., Chicago, IL, USA). Data are expressed as the mean ± standard deviation.

## Results

### *In vitro *IFN-α production is reduced in PBMCs from SLE patients

**Figure 1:**
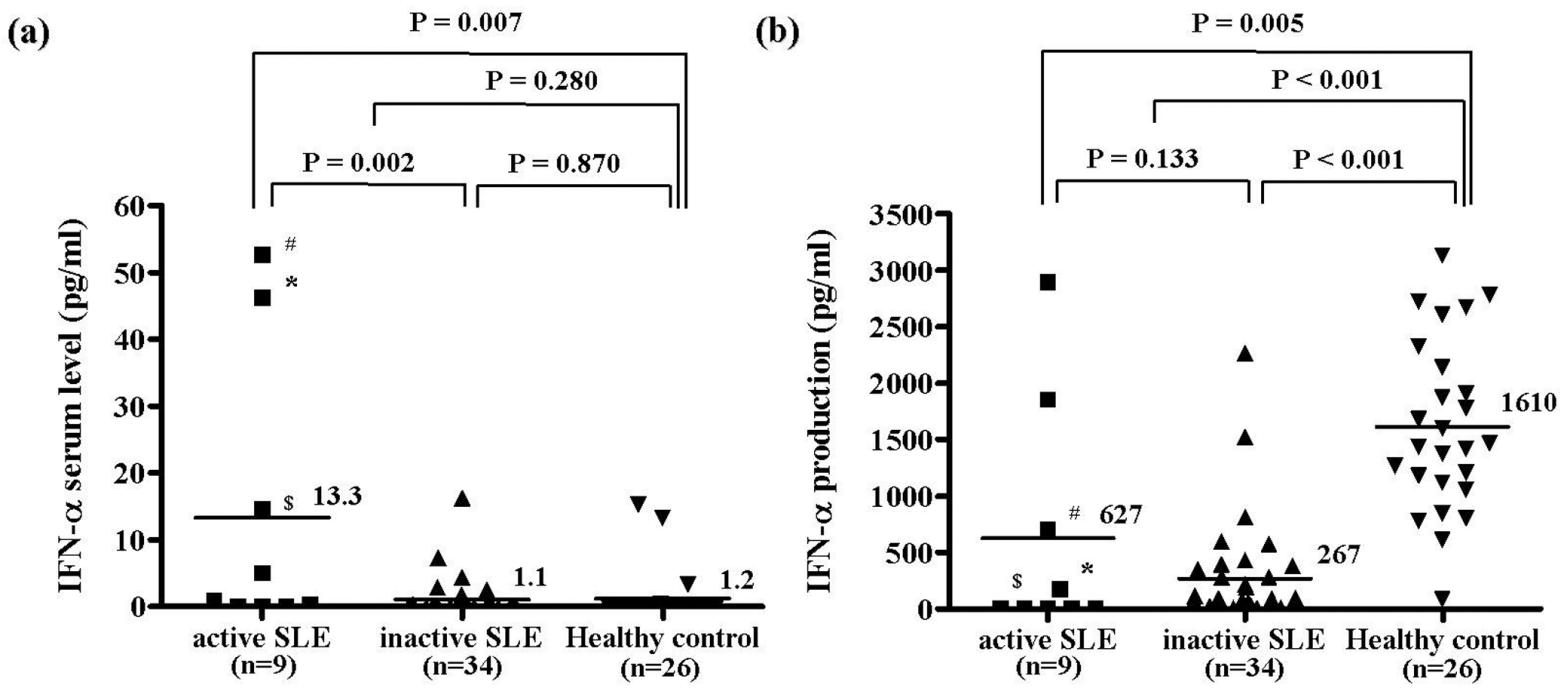
IFN-α serum levels and *in vitro *IFN-α production after CpG ODN2216-stimulation. **(a) **Interferon (IFN)-α serum levels were measured in blood samples collected from patients with systemic lupus erythematosus (SLE) and healthy control individuals using ELISA. Patients with a SLE Disease Activity Index above 4 were classified as having active SLE. Although serum IFN-α levels were higher in patients with active SLE than in those with inactive SLE and healthy control individuals, IFN-α levels in all SLE patients combined were not significantly different from those in healthy control individuals (3.72 ± 3.89 pg/ml in SLE patients [n = 43] versus 1.2 ± 3.9 pg/ml in healthy control individuals [n = 26]; *P *= 0.280 < 0.001). **(b) **IFN-α concentrations in culture supernatants were measured using ELISA after 24 hours of incubation with CpG ODN2216. IFN-α production in peripheral blood mononuclear cells isolated from SLE patients was significantly lower than that in cells isolated from healthy control individuals (342.46 ± 636.82 pg/ml in SLE patients [n = 43] versus 1,610.35 ± 759.56 pg/ml in healthy controls [n = 26]; *P *< 0.001). IFN-α production in peripheral blood mononuclear cells isolated from active SLE patients was also significantly lower than in cells isolated from healthy control individuals (*P *= 0.005). The solid bars represent the mean value in each experimental group. Patients with high IFN-α serum levels are indicated by #, *, and $ in both experiments. Statistical significance was analyzed using Student's *t*-test.

We used ELISA to compare serum concentrations of IFN-α between SLE patients and healthy control individuals (Figure 1a). Patients with active SLE (SLEDAI > 4) exhibited significantly higher levels of IFN-α than did patients with inactive SLE and healthy control individuals (*P *= 0.002 and *P *= 0.007, respectively). However, the levels of IFN-α in total SLE patients (active and inactive) versus healthy control individuals were not significantly different (*P *= 0.280). No correlation was observed between serum IFN-α levels and the clinical characteristics of SLE, such as disease duration, or medications such as steroids and hydroxychloroquine (data not shown).

Because the number of circulating pDCs is very low among total PMBCs, making it difficult to isolate this cell type from a blood sample, IFN-α production was measured in total PBMCs using a pDC-specific TLR9 ligand [20]. PBMCs from patients with SLE and healthy controls werestimulated *in vitro *for 24 hours using the TLR9 ligand CpG ODN2216. IFN-α production was then measured using ELISA. CpG DNA induced IFN-α production was markedly reduced in PBMCs from SLE patients as compared with PBMCs from healthy control individuals (342.46 ± 636.82 pg/ml in SLE patients versus 1610.35 ± 759.56 pg/ml in healthy control individuals; *P *< 0.001; Figure 1b). In addition, IFN-α production was slightly higher in patients with active SLE than in those with inactive SLE, but the difference was not significant (*P *= 0133). No correlation was observed between serum levels of IFN-α and CpG-induced IFN-α production *in vitro *in patients with active SLE. Interestingly, IFN-α production was completely abolished in PBMCs from one-third of SLE patients. These data are almost identical to those reported previously [18]. Although CpG ODN2216 was not used in that previous work, CPG oligonucleotides, herpesviruses, and DNA-containing immune complexes are all TLR9 ligands. Our findings are in accordance with thosse of previous studies showing that PBMCs from SLE patients have reduced capacity to produce IFN-α in response to TLR9 stimulation [16,17].

### Decreased IFN-α production in SLE patients cannot be fully explained in terms of decreased numbers of circulating pDCs

**Figure 2:**
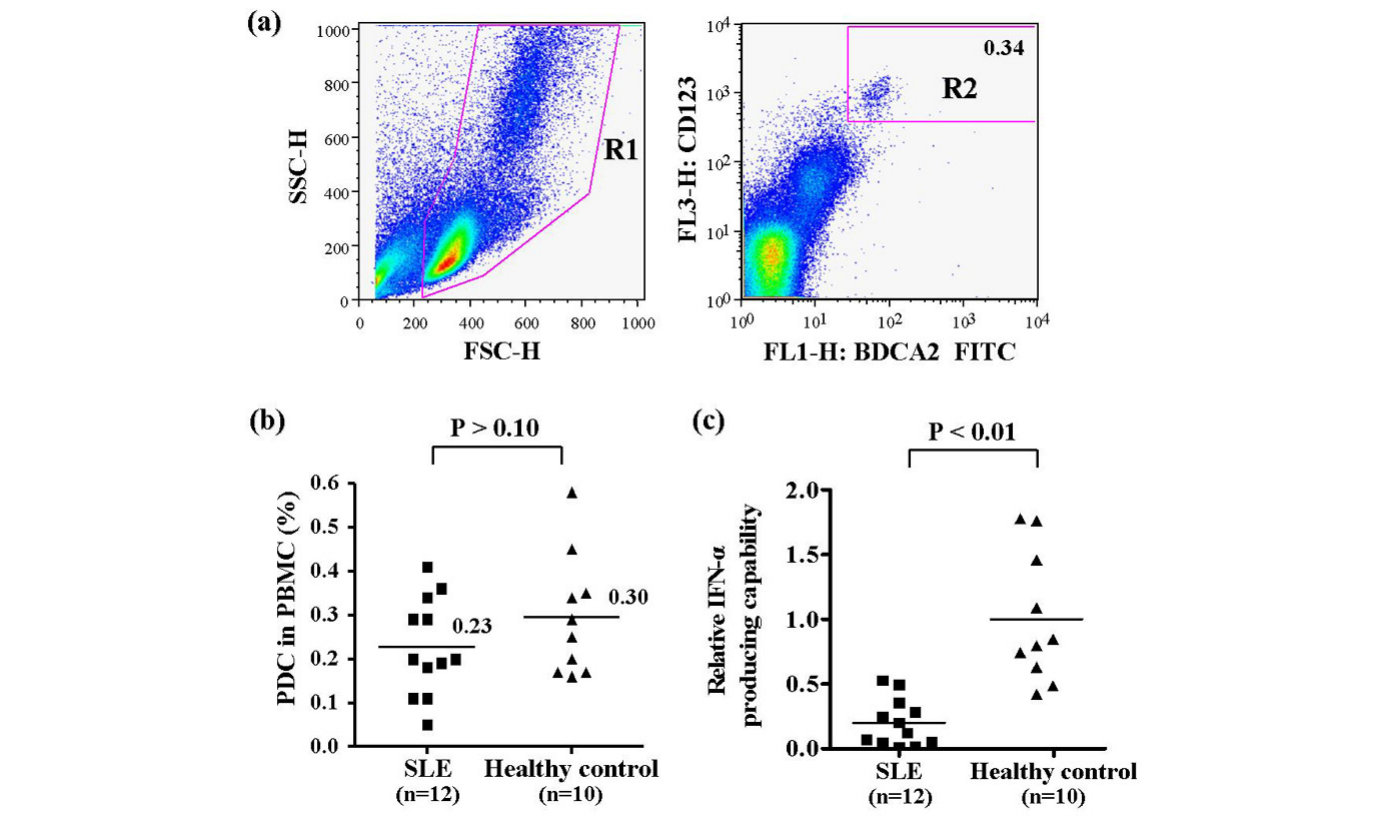
Proportion of pDCs and IFN-α production capacity. **(a) **A gating strategy was used to distinguish plasmacytoid dendritic cells (pDCs) from among peripheral blood mononuclear cells (PBMCs). Blood cells were analyzed using flow cytometry. Total PBMCs were gated at R1 and then analyzed for the presence of blood dendritic cell antigen (BDCA)-2 and CD123. Both BDCA-2-positive and CD123-positive cells were identified as pDCs in gate R2. **(b) **The proportion of pDCs among PBMCs in patients with systemic lupus erythematosus (SLE) versus healthy control individuals. The solid bars represent the mean percentage of pDCs among PBMCs (0.23% in SLE patients versus 0.30% in controls; *P *> 0.10). Statistical significance was analyzed using the Mann-Whitney U-test. **(c) **Relative IFN-α producing capacity in SLE patients versus healthy control individuals. CpG ODN2216-induced IFN-α production was divided by the absolute number of pDCs. **(d) **Expression of Tll-like receptor (TLR)9 mRNA. TLR-9 expression in PBMCs was measured using semiquantitative reverse transcription PCR. The expression of TLR9 is presented relative to β-actin expression. Each analysis was performed in triplicate, and the average values are indicated by a solid square for SLE patients and solid triangle for healthy control individuals. The solid bars represent the mean value for each experimental group. Statistical significance was analyzed using the Mann-Whitney U-test.

Because pDCs are major producers of IFN-α, decreased IFN-α levels in SLE patients may be the result of a drop in pDC count [16,17,23]. To test this possibility, we stained PBMCs with anti-BDCA-2 and anti-CD123, which recognize specific surface markers for human pDCs [23]. During fluoresence-activated cell sorting analysis, PBMCs were selected in gate R1 and BDCA-2^+^/CD123^+ ^cells from gate R1 were deemed pDCs, as shown in gate R2 (Figure 2a). The percentage of pDCs among PBMCs was slightly reduced in SLE patients compared with healthy control individuals (0.23 ± 0.11% in SLE patients versus 0.30 ± 0.14% in controls; *P *> 0.10; Figure 2b). Although this difference was not statistically significant, a decrease in pDC count may partially contribute to reduced IFN-α production. We observed similar results for the absolute number of circulating pDCs (data not shown). Because pDCs are a major source of IFN-α in human PBMCs [10-12], we calculated the relative IFN-α producing capacity of pDCs *in vitro *as follows. The amount of IFN-α produced upon CpG ODN2216 stimulation *in vitro *was divided by the absolute number of pDCs. As expected from results presented in Figures 1b and 2b, the relative IFN-α producing capacity of pDCs was significantly lower in SLE patients than in control individuals (0.2 ± 0.18 in SLE patients versus 1 ± 0.5 in control individuals; *P *< 0.01; Figure 2c). These data, and the fact that PBMCs from one-third of SLE patients did not produce IFN-α, suggest that the observed decrease in IFN-α production is caused by aberrant function in SLE pDCs or depletion of some proportion of pDCs, and not by a decrease in total pDC count. However, there is no way to determine the proportion of pDCs in the blood.

Downregulation of TLR9 may also contribute to decreases in IFN-α production in SLE patients. We used semiquantitative reverse transcription PCR to determine the expression of TLR9 in PBMCs. Interestingly, TLR9 expression was higher in SLE PBMCs than in cells from healthy control individuals (Figure 2d). These data demonstrate that the decrease in IFN-α production was not caused by downregulation of TLR9 expression.

### CpG-induced IFN-α production in SLE PBMCs is inversely correlated with SLE serum induced IFN-α production in healthy PMBCs

**Figure 3:**
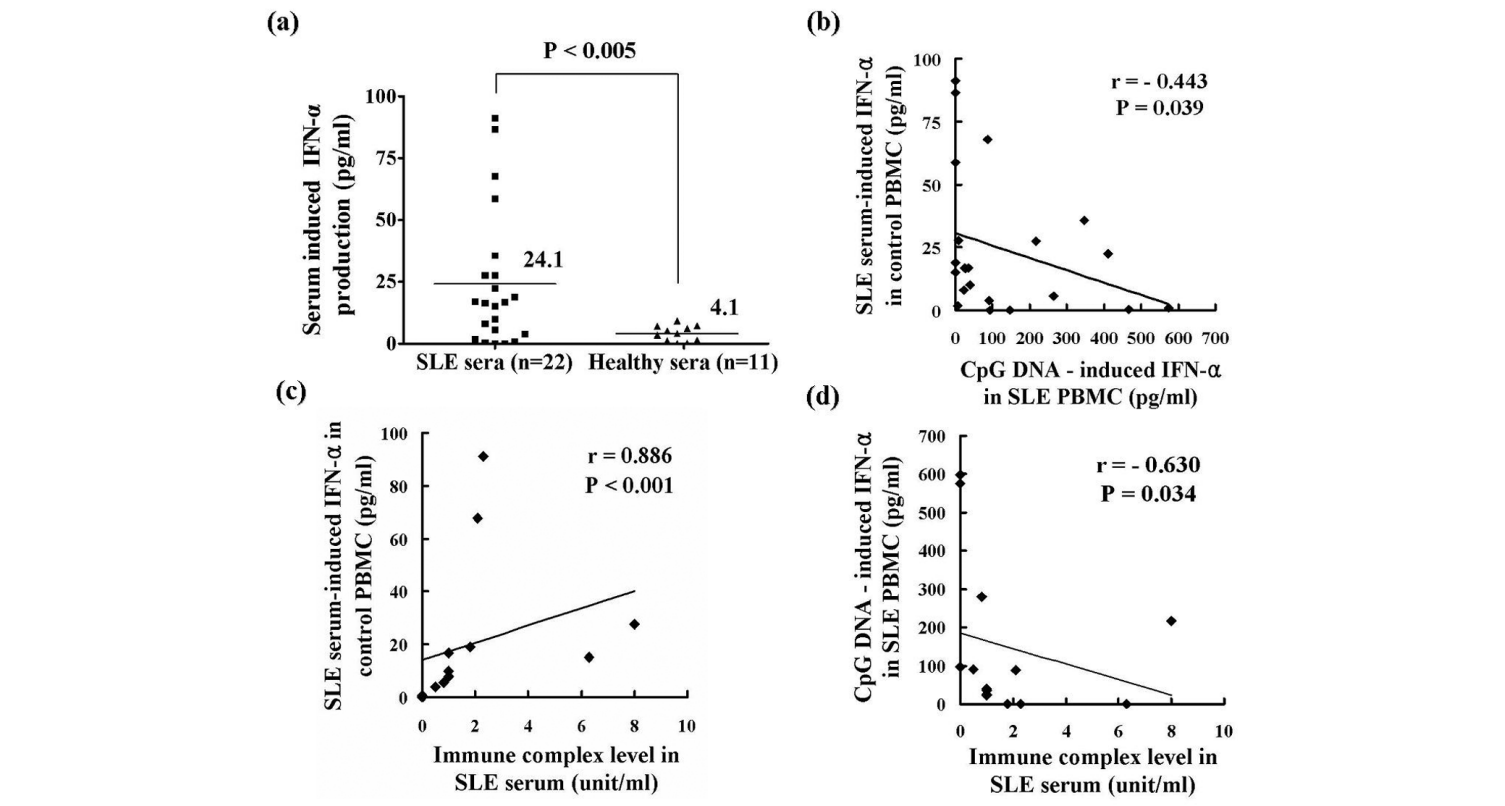
IFN-α production in PBMCs stimulated using serum from SLE patients or healthy control individuals. **(a) **Peripheral blood mononuclear cells (PBMCs), obtained from healthy individuals, were incubated with serum from patients with systemic lupus erythematosus (SLE) or healthy control individuals for 24 hours. Interferon (IFN)-α production was measured using ELISA. The solid bars represent the mean value for each experimental group. The experiment was performed twice using PBMCs obtained from separate healthy individuals. Data are representative of two independent experiments. **(b) **Inverse correlation between the amount of CpG induced IFN-α production in SLE PBMCs (n = 22) versus SLE serum induced IFN-α in control PBMCs. **(c) **Positive correlation between the amounts of immune complexes versus SLE serum induced IFN-α in control PBMCs (n = 14). **(d) **Inverse correlation between the amount of CpG-induced IFN-α production in SLE PBMCs versus the amounts of immune complexes (n = 14). Correlation analyses were performed using Spearman's rank correlation test.

Differences in the numbers of circulating pDCs do not fully explain the difference in IFN-α production capability between the SLE and healthy PBMCs. Furthermore, it has been reported that TLR9 expression levels in the pDCs of SLE patients and healthy control individuals are similar [24]. We suspected that the presence of DNA-containing immune complexes, which function as TLR9 ligands, affect pDC function. In SLE patients, these immune complexes stimulate pDCs to produce IFN-α via TLR9 and FcγRIIa (CD32) [14,15]. We incubated healthy PBMCs with serum from SLE patients and healthy control individuals, and then measured the production of IFN-α. As expected, serum from SLE patients induced IFN-α production to a much greater degree than did serum from healthy control individuals (24.1 ± 27.5 pg/ml versus 4.1 ± 3.14 pg/ml, respectively; *P *< 0.005; Figure 3a). Paradoxically, SLE patients whose serum induces low levels of IFN-α production in PBMCs from healthy individuals possess PBMCs that exhibit higher IFN-α production when stimulated by the TLR9 ligand CpG ODN2216 (Figures 1b and 3b). *Vice versa*, those patients with SLE whose serum induces high levels of IFN-α production in PBMCs from healthy individuals possess PBMCs that exhibit lower IFN-α production in response to CpG ODN2216. In other words, the degree of CpG-induced IFN-α production in SLE PBMCs correlated inversely with SLE serum induced IFN-α production in healthy PBMCs. Also, SLE serum induced IFN-α production in healthy PBMCs correlated with the amount of immune complexes in SLE serum (Figure 3c), and the degree of CpG-induced IFN-α production in SLE PBMCs was inversely correlated with the amounts of immune complexes in matched SLE patients (Figure 3d).

### Repeated stimulation induces TLR9 tolerance in pDCs

**Figure 4:**
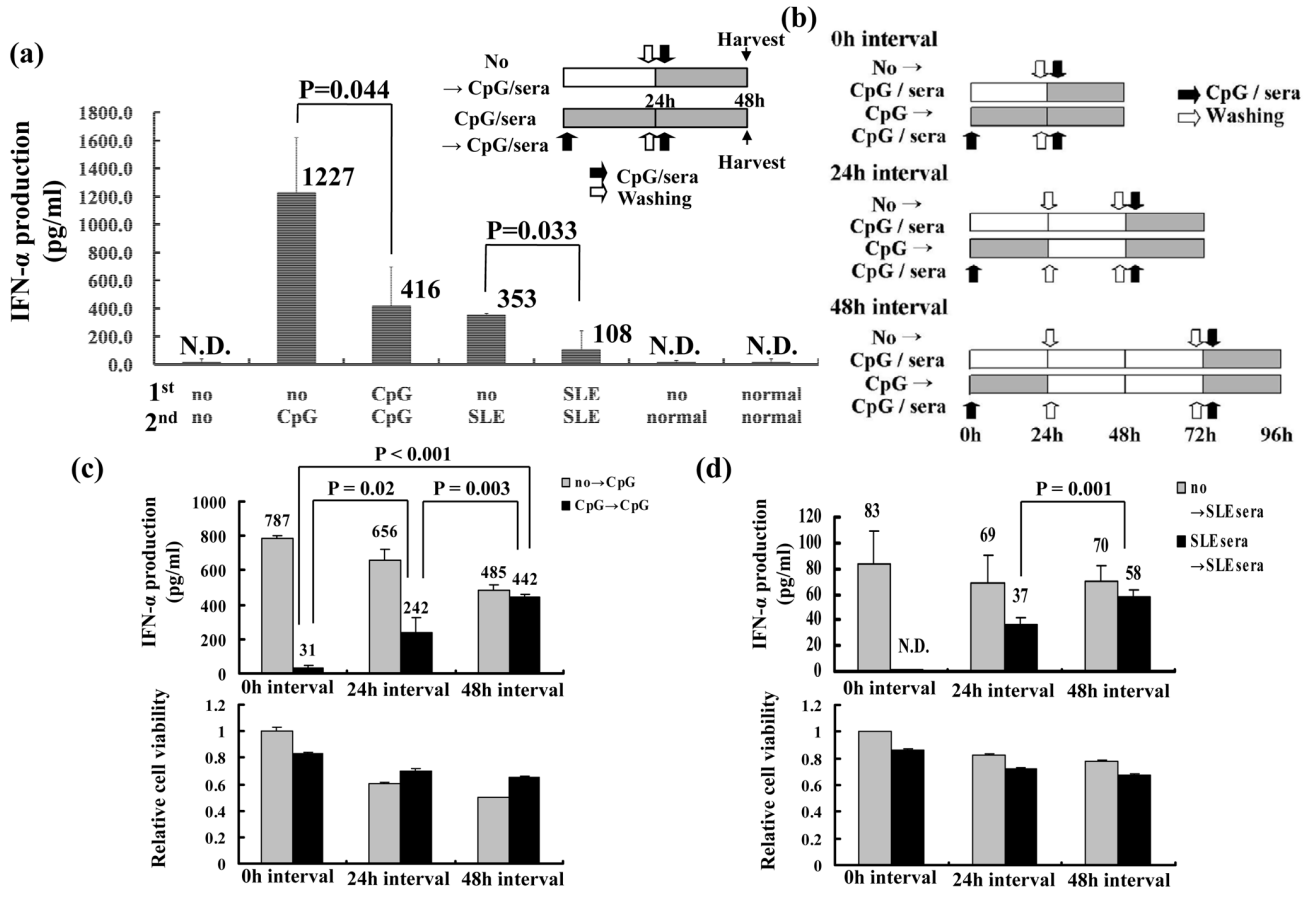
TLR9 tolerance in pDCs. **(a) **Repeated treatment with CpG ODN2216 and systemic lupus erythematosus (SLE) serum reduced interferon (IFN)-α production. Plasmacytoid dendritic cells (pDCs) were purified from peripheral blood mononuclear cells (PBMCs) of healthy individuals using Diamond Plasmacytoid Dendritic Cell Isolation Kit (Miltenyi Biotec) and 2 × 10^4 ^pDCs were incubated with or without CpG ODN2216 or 30% SLE serum. After 24 hours pDCs were carefully washed in serum-free medium and then incubated again with or without CpG ODN2216 or 30% SLE serum. After 24 hours, IFN-α production was measured using ELISA. The experiments were performed in duplicate and three independent experiments were performed using PBMCs from different individuals. The data are presented in (Additional file 1 [Supplementary Figure 4]) and the averages of data are shown. **(b) **Experimental design to investigate the time-dependent recovery of Toll-like receptor (TLR)9 sensitivity. pDCs were purified from total PBMCs of healthy individuals, and 2 × 10^4 ^pDCs were treated with or without CpG ODN2216 or SLE sera for 1 day. The pDCs were then washed with serum-free medium and re-treated with or without CpG ODN2216 or SLE sera for 0 hours, 24 hours, and 48 hours. After 24 hours of treatment, IFN-α production was measured using ELISA. The white and black arrows represent washing and treatment with CpG ODN2216 or 30% SLE sera, respectively. The shaded areas indicate cultures undergoing stimulation. TLR9 tolerance is reversible over time. Purified pDCs were retreated with **(c) **CpG ODN2216 and **(d) **30% SLE serum, as shown in panel b. IFN-α production and cell viability were measured 24 hours after the final stimulation. Each group was duplicated in every experiment and the values shown are the averages of duplicate samples. Three independent experiments were performed using PBMCs from different individuals. One representative case is shown here and the other data are shown in Additional file 1 (Supplementary Figure 5).

Because there was an inverse correlation between IFN-α producing capacity in SLE PBMCs and SLE serum induced IFN-α production in control PBMCs, we hypothesized that the persistent presence of DNA-containing immune complexes may desensitize pDCs to TLR9 stimulation. TLR tolerance, in which cells exhibit no response to subsequent challenges with the same TLR stimulus, has been reported in epithelial cells, neutrophils, macrophages, and conventional dendritic cells [25-28]. To test this hypothesis, we induced TLR9 tolerance by repeatedly stimulating pDCs isolated from healthy individuals using CpG ODN2216 or 30% serum derived from SLE patients. Both IL-3 and granulocyte-monocyte colony stimulating factor are essential cytokines for pDC survival and were thus included in the culture medium [29]. Repeated stimulation using CpG ODN2216 or 30% serum from SLE patients resulted in 65% and 90% decreases in IFN-α production, respectively (Figure 4a); cell viability was not affected (data not shown). These data indicate that repeated stimulation induces TLR9 tolerance in pDCs and are similar to previous data indicating that pDCs produce large amounts of IFN-α within the first 12 hours of activation and become refractory to subsequent stimulation [30].

TLR9 tolerance may be the result of negative feedback in the TLR signaling pathway [31]. Therefore, we examined whether TLR9 tolerance is reversible. pDC PBMCs from healthy individuals were incubated with CpG ODN2216 or 30% SLE serum for 1 day, washed, and then re-stimulated with CpG ODN2216 or 30% SLE serum after a given interval (0, 24, or 48 hours), as indicated in Figure 4b. IFN-α production was measured 24 hours after re-stimulation. IFN-α production decreased upon repeated stimulation at the 0 hours interval and recovered within 48 hours of the first stimulation in repeatedly stimulated pDCs (Figure 4c,d). The cell viability did not differ significantly between stimulation and re-stimulation groups (lower panels of Figure 4c,d). Although we could not test the recovery of IFN-α production capability by pDCs purified from SLE patients, the PBMCs from SLE patients recovered IFN-α production capability over time *in vitro*, without further stimulation, as shown in Additional file 1 (Supplementary Figure 2). These data indicate that TLR9 tolerance is reversible over time and that continuous TLR9 stimulation is required to induce persistent nonresponsiveness in pDCs.

### SLE PBMCs are exposed to IFN-α to a greater degree

**Figure 5:**
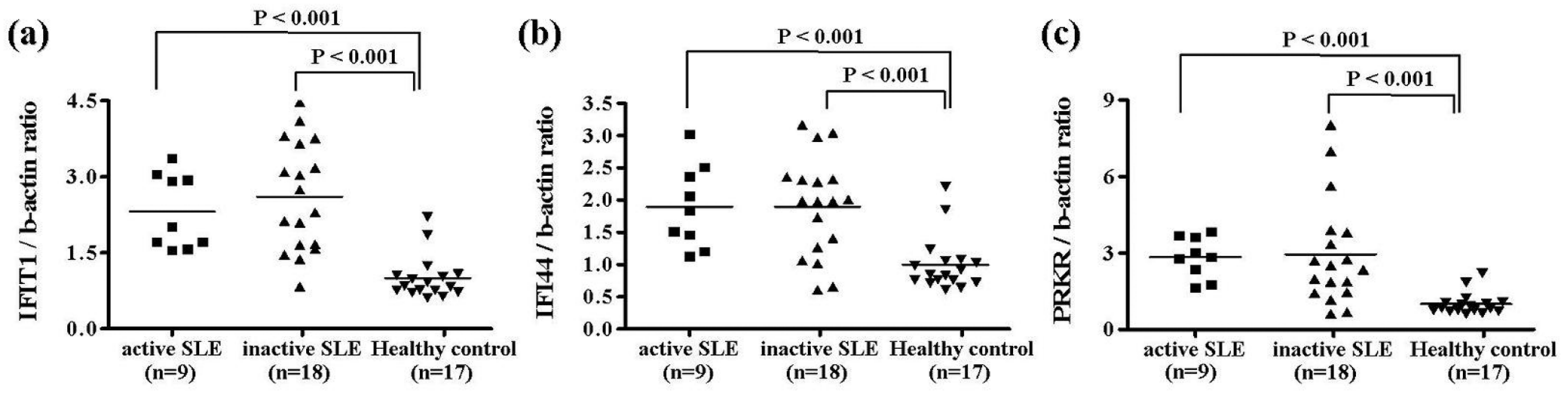
Expression of IFN-α signature genes. Expression of the interferon (IFN)-α responsive genes **(a) ***IFI44*, **(b) ***IFIT1*, and **(c) **PRKR was assessed using semiquantitative reverse transcription PCR. Peripheral blood mononuclear cells (PBMCs) were isolated from patients with systemic lupus erythematosus (n = 27; 9 with active and 18 with inactive disease) and healthy control individuals (n = 17). The expression of each gene is presented relative to β-actin expression. The solid bars represent the mean value for each experimental group. Statistical significance was analyzed using Student's *t*-test.

Because the overall amounts of IFN-α in SLE sera were not significantly different from those in sera from healthy control individuals (Figure 1a), it was unclear whether circulating pDCs had already become TLR9 tolerant. Therefore, we used fluorescence-activated cell sorting to analyze the expression of CD80, CD86, and HLA-DR, which are markers of pDC maturation and activation. However, the number of circulating pDCs was too low to provide conclusive evidence regarding the activation of PDCs. Instead, we examined the expression of IFN-α signature genes in SLE PBMCs [32], including *IFIT1 *(IFN-induced protein with tetratricopeptide repeats 1), *IFI44 *(IFN-induced protein 44), and *PRKR *(IFN-inducible double-stranded RNA-dependent protein kinase). The expression of all three genes was elevated in SLE PBMCs compared with healthy PBMCs (Figure 5a–c). These data imply that SLE PBMCs had been exposed to IFN-α to a greater degree, even though IFN-α levels did not differ between the sera of SLE patients and those of healthy control individuals.

## Discussion

We demonstrated that TLR9-induced IFN-α production is reduced in PBMCsPMBCs from SLE patients (Figure 1 and Table 1). SLE patients exhibit ongoing IFN-α production, and IFN-α serum levels are closely correlated with SLE disease activity [8,9]. Although active SLE serum contains slightly increased levels of IFN-α as compared with inactive SLE and healthy control sera, there were no significant differences in serum levels between total or inactive SLE patients and healthy control individuals. Because some chronic SLE patients had lymphophenia (data not shown), the number of pDCs per blood unit was reduced in SLE patients, which would affect the serum levels of IFN-α. The proportion of circulating pDCs was slightly reduced in SLE patients, and IFN-α production was markedly impaired after *in vitro *stimulation with TLR9, regardless of disease activity (Figures 1b and 2b). Although SLE patients exhibited a slight, nonsignificant decrease in the proportion of pDCs to total PBMCs, we cannot exclude the possibility that this decrease contributes to the decrease in TLR-induced IFN-α production in SLE patients, because the composition of pDC subtypes may differ between SLE patients and healthy control individuals. There is evidence that the Ly6C/Ly49Q pDC subtypes are effective producers of IFN-α [33], and so further investigation is required to determine the composition of pDC subtypes in SLE patients. Other studies have reported that SLE patients exhibit a reduced number of BDCA-2 expressing pDCs, and that herpes virus induced IFN-α production is decreased in SLE PBMCs [16]; furthermore, CpG-induced IFN-α secretion was significantly reduced in monocytes and dendritic cells from SLE patients [18]. However, CpG-induced IFN-α production was completely abolished in one-third of SLE patients, and the decrease in IFN-α production was more marked than the decrease in pDCs, indicating that a different mechanism is at play.

SLE patients exhibited decreased numbers of circulating pDCs (Figure 2b), which is consistent with the findings of a number of other studies [16,23,34], but they also showed increased numbers of pDCs in cutaneous lesions [35,36]. It has been suggested that circulating pDCs are low in SLE patients because this cell type is recruited from the blood to peripheral tissues. However, the fate of circulating pDCs after activation by DNA-containing immune complexes, which present in the blood of SLE patients, is not yet clear. Our results showed that significant numbers of pDCs are still present in the PBMC fraction isolated from SLE patients. Furthermore, we demonstrated that TLR-tolerant pDCs can recover over time and restore IFN-α production (Figure 4c,d), suggesting that pDCs in SLE patients are still present but inactive as a result of TLR tolerance or exhaustion.

The marked decrease or abrogation of IFN-α production may be explained by factors other than cell count. We noted that CpG-induced IFN-α production in SLE PBMCs was inversely correlated with SLE serum-stimulated cytokine production in healthy PBMCs (Figure 3b). We also found that repeated or chronic stimulation of TLR9 by appropriate ligands, such as CpG ODN 2216 or DNA-containing immune complexes, leads to TLR tolerance in pDCs. Although the mechanism of TLR tolerance has not been fully explained, it is a well known occurrence for cells that have been persistently stimulated with TLR ligands to fail to respond to re-stimulation [31,37]. One possible mechanism is inhibition of TLR signaling via dysregulation of lipopolysaccharide-induced TLR4-MyD88 complex formation and IL-1 receptor-associated kinase (IRAK)-1 activation in endotoxin-tolerant cells [38]. Another possibility is induction of genes that negatively regulate TLR signaling, such as IRAK-M and suppressor of cytokine signaling (SOCS)-1 [31,37,39].

We found an increase in the expression of IFN-α signature genes, indicating that SLE PBMCs have already been exposed to IFN-α, which is mainly produced by pDCs (Figure 5). Although we did not check the expression levels of molecules that inhibit the TLR signaling cascade in pDCs from SLE patients, the SLE PBMCs showed elevated levels of IRAK-M and MyD88s compared with the healthy PBMCs (Additional file 1 [Supplementary Figure 3a,b]). Because inflammation may also increase the expression of TLR signaling molecules, we examined the expression of MyD88, which is a positive regulator of the TLR signaling pathway. MyD88 expression was also slightly elevated in SLE PBMCs (Additional file 1 [Supplementary Figure 3c]), although the ratio of MyD88s to MyD88 indicated that the negative regulator, MyD88s, was dominantly expressed in the SLE PBMCs (Additional file 1 [Supplementary Figure 3d]). Although these data do not reveal the functional status of the pDCs in SLE patients, they suggest that the expression of negative regulators of TLR signaling may be responsible for the development of TLR tolerance in the PBMCs of SLE patients. In addition, dysfunctional IFN-α production by SLE pDCs can be induced by other TLR ligands that are found frequently in SLE sera, such as RNA-containing immune complexes and heat shock proteins. However, our investigation was hampered by the limited number of pDCs that could be isolated from the available blood sample, and thus the exact mechanism of TLR-9 tolerance remains to be elucidated. Further investigation is required to clarify this issue.

Another possible mechanism for TLR tolerance is that SLE medications may affect the function of pDCs. Although no correlations were observed among serum IFN-α levels, CpG-induced IFN-α production *in vitro*, and the type and dosage of medicines taken by SLE patients (data not shown), the immuno-suppressors, such as cyclosporine and hydroxychloroquine, can affect the function of pDCs. Hydroxychloroquine, in particular, is a known inhibitor of TLR9 signaling; this drug blocks the acidification of endosomes (phagosomes), which is essential for TLR9 signaling [40]. To rule out the effect of hydroxychloroquine, pDCs from healthy individuals were pretreated with 1 mmol/l hydroxychloroquine for 24 hours, washed twice in serum-free medium, and then treated with CpG ODN2216. After 24 hours of incubation, IFN-α production decreased by up to 60% compared with non-pretreated pDCs (data not shown). These findings indicate that the residual amounts of hydroxychloroquine in pDCs from SLE patients may contribute to TLR tolerance. Moreover, we cannot exclude the potential influences of other medications on pDC numbers and functions. However, not all SLE patients were taking hydroxychloroquine, and the inhibition of TLR9 by residual hydroxychloroquine cannot fully explain the abrogation of IFN-α observed in one-third of SLE patients (Figure 1).

## Conclusion

In the present study we demonstrated that circulating pDCs are desensitized to TLR9 stimulation in patients with chronic SLE. This desensitization is probably the result of persistent stimulation by DNA-containing immune complexes, which are a hallmark of SLE. In SLE patients, pDCs become tolerant to TLR9 stimulation or exhausted in terms of IFN-α production. These findings provide important insight into the pathogenesis of SLE and the markedly increased incidence of certain viral infections in SLE patients. In addition, our data indicate that the role of IFN-α is different in developing SLE and in chronic SLE.

## Abbreviations

BDCA = blood dendritic cell antigen; ELISA = enzyme-linked immunosorbent assay; IFN = interferon; IL = interleukin; IRAK = IL-1 receptor-associated kinase; PBMC = peripheral blood mononuclear cell; PCR = polymerase chain reaction; pDC = plasmacytoid dendritic cell; SLE = systemic lupus erythematosus; SLEDAI = SLE Disease Activity Index; TLR = Toll-like receptor.

## Competing interests

The authors declare that they have no competing interests.

## Authors' contributions

J-YL and S-KK carried out the experimental work, performed the statistical analysis, and drafted the manuscript. Se-Ho P analyzed and interpreted data. M-LC and S-YM performed statistical analysis. Sung-Hwan P provided patient's blood samples. Y-GC and H-YK designed and conceived of the study, coordinated the project, and drafted the manuscript. All authors read and approved the final manuscript.

## Supplementary Material

Additional file 1 Additional file 1 is a Word file that contains Supplementary Figures. Click here for file
