# Supplementary material for: Dysfunctional interferon-α production by peripheral plasmacytoid dendritic cells upon Toll-like receptor-9 stimulation in patients with systemic lupus erythematosus
Source: Arthritis Res Ther. 2008 Mar 6;10(2):R29. doi: 10.1186/ar2382 (PMC2453773; doi:10.1186/ar2382)
Supplement: Additional file 1 — is a Word file that contains Supplementary Figures. [file ar2382-S1.doc]

**Supplemental figure 1**

**A representative standard curve for the IFN-α ELISA**

**Supplemental figure 2**

**The IFN-α production capability of SLE PBMCs recovered over time, without further stimulation in vitro.** PBMCs (1×10^6^) isolated from SLE patients (a) and healthy individuals (b) were incubated with CpG ODN2216 at 0 h, 24 h, 48 h, and 72 h after isolation. IFN-α production was measured by ELISA 24 h after each treatment. Each group was assayed in triplicate and the solid bars represent the mean value for each experimental group. The experiment was performed twice using PBMCs obtained from different SLE patients and healthy individuals. The data shown are representative of two independent experiments.

**Supplemental figure 3**

**Expression of factors involved in the TLR signaling pathway.** The expression of the TLR signaling molecules, (a) MyD88s, (b) IRAK-M, and (c) MyD88, were examined in PBMCs from SLE patients (n = 27 ; active = 9, inactive = 18) and healthy controls (n = 17) using semi-quantitative RT-PCR. The expression of each gene is presented relative to β-actin expression. (d) Ratio of MyD88s/MyD88 expression. The solid bars represent the mean value for each experimental group. Statistical significance was analyzed using Student’s *t*-test.

**Supplemental figure 4**

**
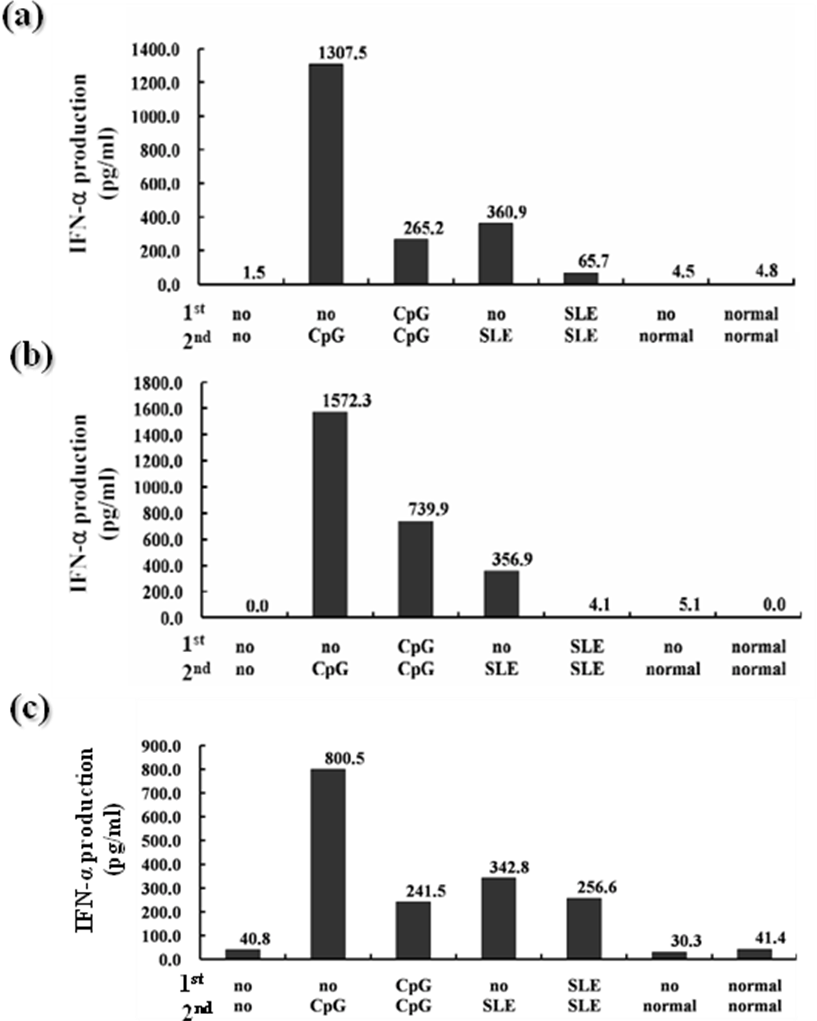
**

**TLR-9 tolerance in pDCs.** (a) Repeated treatment with CpG ODN2216 and SLE serum reduced IFN-α production. pDCs were purified from PBMCs of healthy individuals using Diamond Plasmacytoid Dendritic Cell Isolation Kit (Miltenyi Biotec) and 2 × 10^4^ pDCs were incubated with or without CpG ODN2216 or 30% SLE serum. After 24 h, pDCs were carefully washed in serum-free medium and then incubated again with or without CpG ODN2216 or 30% SLE serum. After 24 h, IFN-α production was measured using ELISA. The experiments were performed in duplicate and three independent experiments were performed using PBMCs from different individuals. Three cases are shown here. The values shown are the averages of duplicate samples.

**Supplemental figure 5**

TLR9 tolerance is reversible over time. pDCs were purified from the total PBMCs of healthy individuals, and 2 × 10^4^ pDCs were treated with or without CpG ODN2216 for 1 day. The pDCs were then washed with serum-free medium and re-treated with or without CpG ODN2216 for 0 h, 24 h, and 48 h. After 24 h of treatment, IFN-α production was measured by ELISA. IFN-α production were measured 24 h after the final stimulation. Each group was duplicated in every experiment and the values shown are the averages of duplicate samples. Three independent experiments were performed using PBMCs from different individuals. One representative case is shown in main articles and two another cases are shown in (a) and (b).
